# Underwater Energy Harvesting to Extend Operation Time of Submersible Sensors

**DOI:** 10.3390/s22041341

**Published:** 2022-02-10

**Authors:** Carlos L. Faria, Marcos S. Martins, Tiago Matos, Rui Lima, João M. Miranda, Luís M. Gonçalves

**Affiliations:** 1 CMEMS-UMinho, Campus de Azurém, University of Minho, 4800-058 Guimarães, Portugal; mmartins@dei.uminho.pt (M.S.M.); matos.tiagoandre@cmems.uminho.pt (T.M.); lgoncalves@dei.uminho.pt (L.M.G.); 2 LABBELS–Associate Laboratory, 4710-057 Braga, Portugal; 3 METRICS-UMinho, Campus de Azurém, University of Minho, 4804-533 Guimarães, Portugal; rl@dem.uminho.pt; 4 CEFT, Transport Phenomena Research Center, University of Porto, 4200-465 Porto, Portugal; jmiranda@fe.up.pt

**Keywords:** LEG, submersible energy harvesting, energy sensors, ocean energy harvesting, linear electromagnetic generator

## Abstract

A linear electromagnetic energy harvesting device for underwater applications, fabricated with a simple manufacturing process, was developed to operate with movement frequencies from 0.1 to 0.4 Hz. The generator has two coils, and the effect of the combination of the two coils was investigated. The experimental study has shown that the energy capture system was able to supply energy to several ocean sensors, producing 7.77 mJ per second with wave movements at 0.4 Hz. This study shows that this energy is enough to restore the energy used by the battery or the capacitor and continue supplying energy to the sensors used in the experimental work. For an ocean wave frequency of 0.4 Hz, the generator can supply power to 8 sensors or 48 sensors, depending on the energy consumed and its optimization.

## 1. Introduction

Advances in electronics have allowed the continuous reduction in the power consumption of sensors and an increase in their autonomy [1,2]. Moreover, the use of small renewable energy sources can sustain the operation of the sensor during its lifetime [3,4,5]. Energy harvesting has been an emerging theme for monitoring sensor networks in which each node is individually autonomous in terms of energy, reducing maintenance and replacing battery costs. To deal with underwater sensor systems and other similar applications, it is necessary to develop autonomous energy harvesters. Various types of energy capture were studied, with different operating principles and materials, to supply energy to the sensors, with different advantages and disadvantages. These devices can be divided into two main categories: semi-submerged devices, which will work on the sea surface; and fully submerged devices, which will work completely below the sea surface.

There are two major barriers to the smooth operation of energy harvesters in sea settings: on the one hand, the aggressiveness of the environment (for example, the impact of waves, navigation, weather conditions and marine fauna) and, on the other hand, the problem caused by biofouling, a phenomenon consisting of macro and microorganisms growing on the device surfaces. Biofouling has significant effects on the structures, reducing device lifetime and performance [6].

The advantages of operating with a device on the surface of the ocean are its easiest maintenance and the diversity of technological options, for example, using solar, wind, electromagnetic, and piezoelectric energy, among others. The disadvantages are that they are subjected to the impacts of the waves, they can cause problems for maritime navigation (there must be a reserved area, a farm, for the devices), marine animals can damage the devices and they are subject to biofouling.

For devices that will work underwater, the main advantages are not being exposed to the impact of the waves, and not being exposed to the sun (thus, UV protection materials and treatments are avoided). As for disadvantages, there is the fact that these cannot use solar energy or wind; there may be algae accumulation in the device; it may be damaged by fishing nets; and problems with biofouling are also present. Despite different applications, common harvesting technologies can be used in these devices.

In piezoelectric technology, much research work was conducted on optimizing designs of piezoelectric energy harvesting systems. In 2007, Zurkinden et al. [7] presented a wave energy device with piezoelectric materials. They used piezoelectric polymers (PVDF), and due to viscous stress and fluid pressure exerted by the wave movement, the piezoelectric polymer bends continually, producing electrical energy. The resulting undulating motion of the system resembles the movement of a sea plant in the ocean ground [7]. However, the amount of energy generated by the studied piezoelectric device was very small. Xie et al., in 2014 [8], presented a device based also on the piezoelectric effect, with large dimensions, with a minimum length of 1.2 m (length depends also on depth). The average power increases non-linearly with the increase in the wave height, and the results obtained by the authors show that with wave heights of 2, 3 and 4 m, the device can produce, respectively, 1.5, 7.4 and 23.3 W of power. This system is conditioned to the height of the waves and has to be positioned close to the surface. Maamer et al. [9] presented a review on the design improvements and techniques for mechanical energy harvesting using piezoelectric and electromagnetic systems. Several designs can eventually be applied into the ocean. Xiaofeng Wang et al. [10], in 2015, reported a triboelectric nanogenerator (TENG), based on a fully enclosed rolling spherical structure with 6 cm in diameter for harvesting low-frequency wave energy that can provide a peak current of 1 μA with an instantaneous output power of up to 10 mW. Other TENG configurations can also be found in the literature [11,12,13]. Tao et al. [14] developed a tribolectric generator inserted in a spheric floating buoy to use on the ocean surface with the use of TEG. This system was inspired in an origami configuration and has the advantage of working with various frequencies and amplitudes as well as in arbitrary directions. This work reported an instantaneous open-circuit voltage and short-circuit current of 1000 V and 110 μA. Li et al. [15] applied in a spheric body a pendulum inspired triboelectric nanogenerator (P-TENG). With this configuration, they reported that it was possible to improve the harvesting efficiency through a transformation of impact kinetic energy into potential energy with the possibility of contributing to the blue energy. This device can be applied to the ocean surface. Zhao et al. [16], in a review about recent progress in blue energy harvesting for powering distributed sensors in the ocean, showed several solutions to work on the sea surface with waves. The majority of the devices shown in this review work with TENG or hybrid systems, TENG and EMH (electromagnetic harvesting). Shen et al. [17] presented recent advances towards ocean energy harvesting and self-powered applications based on triboelectric nanogenerators and the study of different materials and configurations. In this review, it was stated that at present, the most employed hybrid generator is the EMG (electromagnetic generator)-TENG, as EMGs increase the current of hybrid generators. The numerical study performed by Kong et al. [18] reported a new energy harvesting system using vortex-induced vibration (VIV). In this work, the authors reported that the vibrations of a bluff body are the result of the alternating vortices created by the unsteady separation. It should be noted that the external body of the energy harvesting system may create the vibration from outside to inside. Wang et al. [19] presented another review about the energy harvesting flow-induced vibration. Some of the presented devices can be applied into the ocean, using ocean tidal energy. However, they reported that the flow mechanism around a complex bluff body surface needs to be further studied.

Fan Shen Reza Valizadeh et al. [20] reported a mini turbine (60 cm in diameter) that works on the sea surface and can produce 1600 W at high frequencies. Another technology that can be used to manufacture a mini- or micro-generator is the electromagnetic effect or combination of the piezoelectric and electromagnetic effects. The electromagnetic systems employ an electromagnetic induction, which is the variation of the magnetic flux, through a conductor that induces a voltage in its ends [9].

Several configurations are possible, with levitation systems [21,22], damping springs [23,24] or high-frequency vibrations [25,26]. Some configurations use springs on top and magnets on the bottom to amplify the damping effect [27]. Another possible combination is to convert the available linear movement to a rotating movement, suitable for an electromagnetic generator, using gears to increase the rotation speed, as reported by Guo et al. [28]. This device works on the surface of the ocean and can produce 122 mW of electric power with a maximum voltage of 4.45 V at 1.0 Hz wave frequency. The total dimension of this device is 100 × 64 × 80 mm. Farrok et al. [29] used a linear conversion device, with flat magnets. To promote the movement of the magnets, a buoy that oscillates with the waves was connected to the magnets.

In submersible applications, it is possible to apply the linear configuration reported by Chiu et al. [30,31]. As described by the authors, the device uses water currents as an energy source. The output power depends on the depth of the seabed. Near the surface, it will generate higher power than near the seabed. This device can produce up to 5.8 mW, with an induced voltage of 1.5 V.

## 2. Energy Harvesting System Description

**Figure 1:**
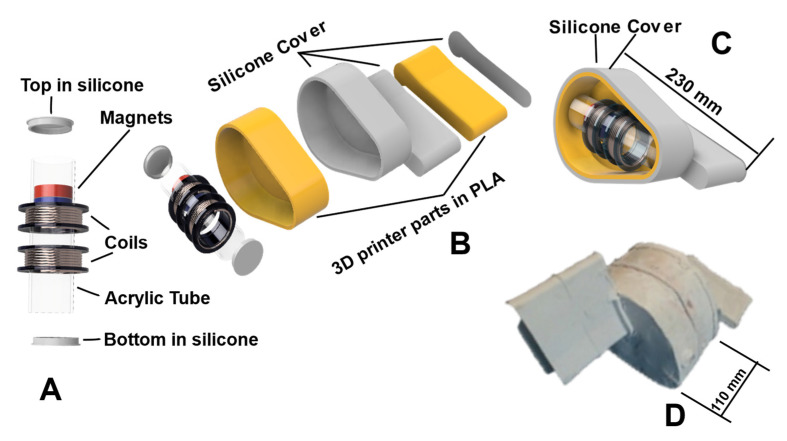
Energy harvesting device. (**A**) LEG, consisting of two copper coils, acrylic tube and silicone stops. (**B**) Body in cut to show the LEG and its components. (**C**) Body assembled in cut. (**D**) Silicone outer shell of LEG.

**Figure 2:**
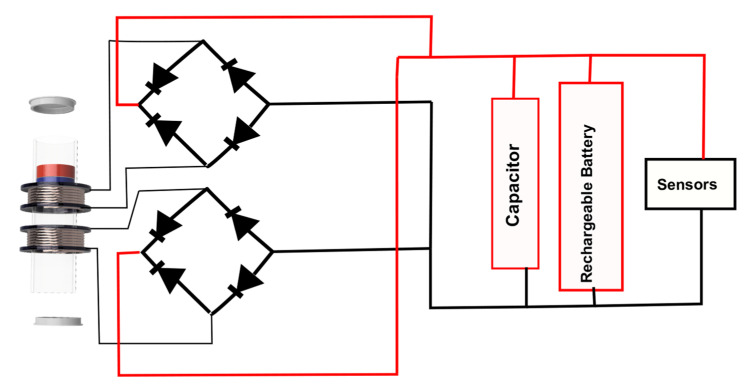
Functional diagram of the implemented energy harvesting system.

An energy harvesting device to work fully submerged is presented in this work. The power capture device is intended to be applied to supply power to submerged sensor networks, in depths from 3 to 10 m, having the advantage of not being directly impacted by the waves, not being vandalized, and avoiding small marine traffic. The device is based on a linear electromagnetic generator (LEG) as presented in Figure 1 and Figure 2. The magnets have a disk shape, selected to keep always the same flux direction, contrarily to the spheres used by Joo et al. [32]. When the sphere rotates around its magnetization axis, the magnetic flux through the coil changes from its maximum value to zero, while disks are always oriented to use a maximum magnetic field.

This configuration was adopted to avoid disadvantages mentioned in previous works from the literature. Any type of movement conversion was avoided, for example, conversion from linear to rotary motion, which would cause lower system efficiency. The electromagnetic generator is inside a silicone body, with no external moving parts, thus preventing malfunction due to biofouling. This option prevents the deterioration of these components and since the device will be completely submerged, ambient UV light protection is dispensable.

The experimental work demonstrates that the energy harvesting device proposed not only supplies power to the sensors but is also able to charge a battery or a capacitor. It is possible to extend the operating time (reducing the time between battery replacements) or even implement an energetically autonomous sensor network, optimizing resources and reducing the environmental impact.

The proposed LEG is placed inside of a body, manufactured in a 3D printed with polylactic acid (PLA) material, and this body is coated with a silicone body designed to capture the mechanical energy (movements from water bodies). Figure 1 shows the outside body and the inside view of the LEG. The exterior body is anchored to the seabed with a rope. The harvester device uses the movement of seawater that occurs vertically, perpendicular to the wave direction to promote the LEG movement, as torque is obtained from the buoyancy force and drag and lift forces due to water flow.

A capacitor of 1F/9 V and 3.7 V/2400 mA LiPo battery is used to store the energy to power the sensors, using energy management of the harvested energy to maximize its use and storage [33,34,35]. A full bridge rectifier is used to perform AC/DC conversion, as presented in Figure 2, to connect the LEG to sensors and charge the storage device. Schottky diodes (BAT54CL) are used in a full-wave bridge rectifier, with an expected forward voltage drop of 0.3 V.

In the experimental device characterized in this work, a capacitor is used, instead of a rechargeable battery. The purpose of using only the capacitor is to allow better visualization and analysis of the stored energy by the charged voltage. The proposed system has LEG, an AC/DC converter, a capacitor and power several sensors. The sensors used in the experimental work are designed for the ocean for turbidity monitoring [35].

## 3. Materials and Methods

The harvester outer structure was optimized to maximize the capture of underwater wave movements and promote the linear electromagnetic generator (LEG) oscillations. The optimization study and fabrication details of this system can be found elsewhere [36,37]. The experimental characterization, including free-fall tests, load dependence and oscillation frequency dependence, is now presented.

### 3.1. The LEG Prototype

**Figure 3:**
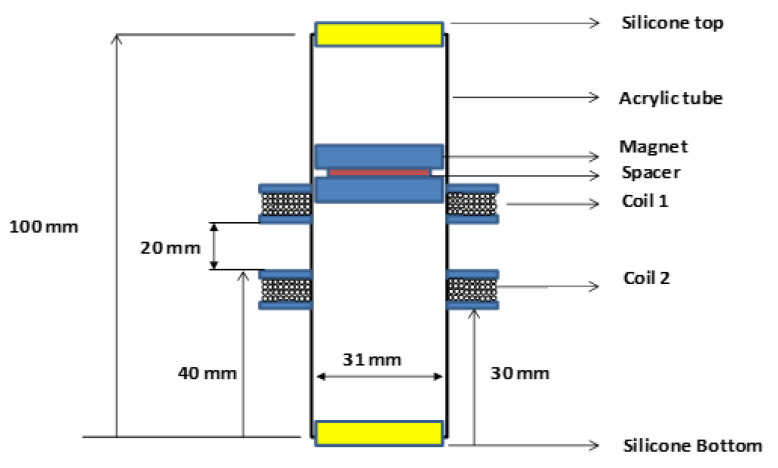
LEG configuration and dimensions.

The experimental LEG (see Figure 3) is composed of two coils, with a gap of 20 mm between them. When external acceleration is applied to the device (i.e., the acrylic tube), the magnet mass moves through coils and an induced voltage is produced in coils terminals. The device does not use springs (on the top or bottom) or damping magnets (levitation effect), but silicone discs with a small hole to promote air exhaustion. This simple configuration reduces the mechanical moving parts (springs) and takes advantage of the longest possible stroke. Air exhaustion is important when the magnet moves inside to avoid a force against the movement since the gap between the magnet and the tube is only 0.5 mm.

The LEG is composed of an acrylic tube, with 100 mm of length, 31 mm of interior diameter and a thickness of 2 mm. The top and bottom ends of the tube include a silicone disk to dampen the final movement of the magnets. Two N45 magnets discs of neodymium iron boron (NdFeB), with 30 mm of diameter and 1.32 T residual magnetism (Br) are placed inside the acrylic tube. The direction of magnetization is axial, and the two magnets are connected with a north–south connection, with a separating non-magnetic disk between them, with a diameter of 26.0 mm and 6 mm of thickness, 3D printed in polylactic acid (PLA) material. The mass of each magnet is 53.7 g.

The coils are placed outside of an acrylic tube, fabricated with isolated copper wire 0.2 mm of diameter, with 1500 turns. The resistance of the coil is around 125 Ω, with an outside diameter of 60 mm and 15 mm in width.

This LEG dimension is used, as it is intended to insert the LEG inside a small body (Figure 1D). The outer body is made of silicone to protect the LEG from seawater and provide energy to the sensors.

Two coils are used, with a distance between them as mentioned in a previous work [36]. It is intended to capture the variation of the magnetic flux in both directions; with the coils near to the silicone disk of the acrylic tube, it is possible also to capture the damping effect with small amplitude. If the springs are used at the ends, eventually, it would not use the entire length of the acrylic tube to promote the magnet movements and, in this case, it would have less kinetic energy. If magnets are used at the ends of the acrylic tube, eventually the magnet will stop most of the time between the magnets, due to the length of the tube, the width of the coils and the distance between the coils. In this situation, there is the dampening effect of the magnets, and the velocity will be compromised. The use of springs or magnets at the ends of the LEG will be advantageous with a longer length of LEG or eventually with high frequencies. In the present work, the device should have the smallest dimensions possible; as a result, frequencies between 01 and 0.4 Hz are used.

### 3.2. Methods

The Portuguese coast is frequently reached by waves formed in storms with the center in the middle of the Atlantic Ocean, as well as waves created by the local wind. Additionally, considering that our device will be installed at depths between 3 and 10 m, close to the coast, swell waves (regular and long waves with crest generated in a storm that travelled a long distance) and wind waves (irregular and short waves generated by the local wind) are considered and analyzed. The values of the periods wave on the Portuguese coast and the interaction between depth and waves, working frequencies between 0.1 and 0.4 Hz, are considered. Another reason to consider these frequencies is that the device will work in regions with waves and current interactions. In the ocean, the device will be submitted to oscillations with various frequencies through the water, and frequency may eventually be influenced by the depth, the proximity of the coast, the morphology of the seabed and the artificial structures existing in the neighborhood. With all these variables, this experimental work is intended to verify whether it is possible to increase the autonomy of the sensors or even make the sensors energetically autonomous. Some authors use mathematical models to predict the values of electrical energy for the device configurations [38,39,40]. In this work, the mathematical expressions are obtained, taking into account the experimental results of the individual coils tests to predict the best configurations, and results are confirmed experimentally with freefall tests, load tests and frequency tests to predict the output electrical power and confirm the calculated values. Finally, the generator is tested, powering several turbidity sensors, simulating a real application.

#### 3.2.1. Freefall Tests

Freefall tests are performed to find out which connection configuration (parallel or series of the two coils) corresponds to the highest electrical output power. The open-circuit voltage is measured since it is directly correlated to the output power. Both parallel or series connections are tested, both connections before and after the full-wave bridge rectifier (FWBR) circuit (AC/DC).

In the freefall tests, the magnet falls freely vertically (without friction) and the output voltages are measured over time. The voltage is measured with a PicoScope 2205A with an 8 kS/s sample rate and 125 µs sample interval. The test is carried out by the magnet falling in the direction of Coil A to Coil B and vice versa to verify the expected symmetry of the device. The purpose of the tests is to verify which connection configuration produces higher voltages, thus higher power, for longer periods. With a direct generator–battery connection, the battery can be charged if the generated voltage is above the battery voltage (a lithium rechargeable battery rises from 3 V when discharged up to 4.1 V when charged). The mean voltage of 4 V is used as the minimum voltage to obtain energy from the generator.

#### 3.2.2. Load Tests

The open-loop output voltage measurement can be used to predict power; however, the output power is obtained only when a load connects to the generator. The effect of the load in the output power, to find maximum output power and operational output power, is measured (when a battery is acting as a constant voltage source directly connected to the generator). Theoretically, the maximum power should be obtained when the load resistor matches the internal resistor of the generator. However, the effect of the drag forces caused by magnet induction with the current generated inside the coils affects the speed of the magnet travelling and, consequently, the output voltage and time delay. To calculate the electric power that the device can supply, a set of resistors is introduced as the load, and the delivered power is calculated.

#### 3.2.3. Frequency Tests

**Figure 4:**
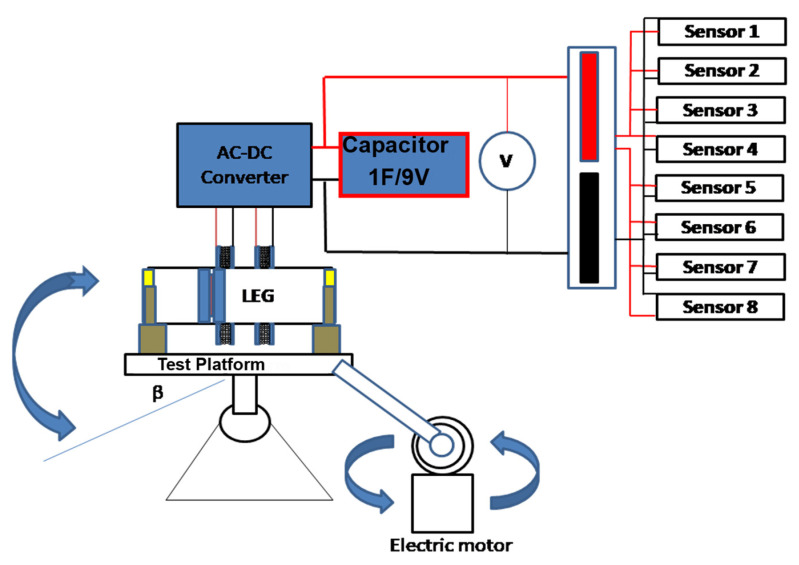
Equipment for testing different frequencies in the LEG.

It is intended for this device to work with small frequencies of typical ocean waves. For this reason, the device is characterized with movement frequencies from 0.1 to 0.4 Hz. To simulate these frequencies, the setup shown in Figure 4 is implemented and used.

The LEG is placed horizontally and fixed to the platform, using two non-metallic fittings. All components of the platform have non-magnetic properties.

The frequencies to be tested are introduced into the test platform by the electric motor, promoting the oscillatory motion, with maximum descending and ascending angle (β) of 45° from the horizontal line of the platform. The 45° was selected for this test because it is above the angle obtained for static friction (15°) between the materials and based on previous work [35].

Using the configuration previously selected, tests are carried out with a set of load resistors, to obtain the output power and energy.

The electrical properties of the sensors [35] (instantaneous power during reading and sleep mode of the sensors, and energy consumption in a complete 300 s cycle) are presented in Table 1. Each sensor, when connected to energy, reads data, each 300 s, and each reading cycle lasts for 10 ms. All sensors enter in sleep mode after doing the readings; the sleep mode time is the same for all sensors, 299.99 s. When several sensors are connected to the same network, readings for each sensor are delayed 2 s from the previous sensor.

## 4. Results and Discussion

With the tests carried out, it is possible to verify if the system is able to supply energy to the sensors and restore the energy supplied by the battery.

### 4.1. Open-Circuit Voltage Output

**Figure 5:**
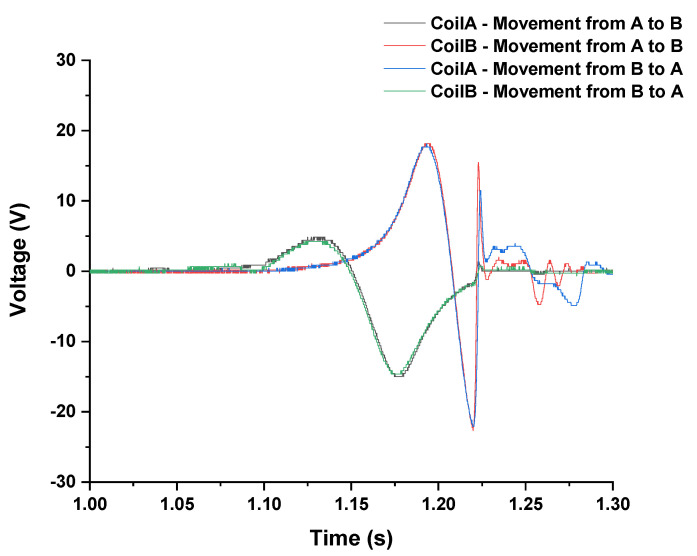
Output voltage of each coil (Coils A and B) in a freefall test, when magnets move in the direction from Coil A to Coil B (A–B) or vice-versa (B–A), the magnets pass through coils in 250 ms.

It is important to find the maximum voltage that the energy harvesting system can deliver to the storage system and how long it will be able to deliver the minimum voltage necessary to allow the system to work since power can be delivered only when the output voltage is higher than the capacitor or battery voltage. The LEG needs to supply at least a voltage superior at 4.0 volts. Figure 5 presents the voltage achieved in freefall tests (average voltage from a set of six tests), with the magnet moving from Coil A to Coil B (A–B) and vice-versa (B–A), for each coil (Coil A or Coil B).

The symmetry of the device is verified. The output voltage of Coil A, when the magnet moves from A to B, is almost equal to the voltage in Coil B when the magnet moves from B to A.

**Figure 6:**
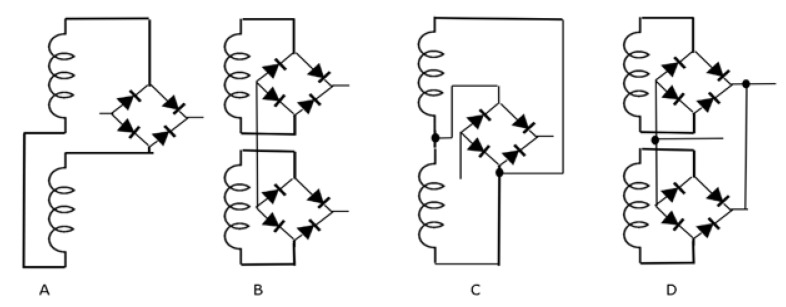
(**A**) Coils in series followed by rectification. (**B**) Coils in series after rectification. (**C**) Coils in parallel followed by rectification. (**D**) Coils in parallel after rectification.

With the experimental output voltage of each coil along time, it is possible to predict the theoretical voltage output values of the configurations (example, series or parallel of coils) that best adapt to the intended system. Four combinations are tested: the rectification of the combined voltages (parallel or series) before passing through the rectifier bridge and the combination of the rectified voltages (parallel or series) after rectification, that is, performing the combination of the voltages after passing each one through the rectifier bridge. To analyze which combination best fits the proposed system, theoretical combinations are carried out before tests. The tests are then carried out to verify the agreement with the theoretical values. Figure 6 presents the four combinations.

#### 4.1.1. Coils in Series Followed by Rectification

Theoretical voltage output when coils are connected in series, followed by rectification, is obtained by Expression (1), where V*n* is the output voltage, V*coilA* and V*coilB* are the experimental voltage values for each coil, obtained in the freefall test.

(1) $$V_{n}=\{\begin{array}{c} \;\;\;\;\;\;\;\;\;\;\;\;\;\;\;\;\;\;\;\;\;\;\;\;\;\;\;\;\;\;\;\;\;\;\;\;\;\;0,\;\;\;\left|V_{CoilA}-V_{CoilB}\right|<0.30\\ \left|V_{CoilA}-V_{CoilB}\right|-0.30,\;\;\;\left|V_{CoilA}-V_{CoilB}\right|\geq 0.30 \end{array}$$

**Figure 7:**
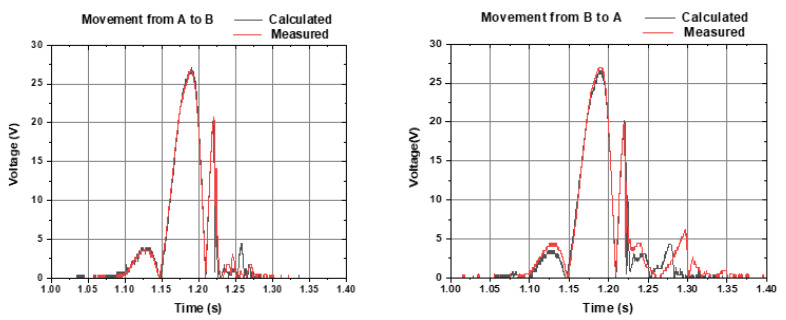
Calculated voltage (grey line) and experimentally measured voltage (red line) of the output, within a freefall movement from Coil A to Coil B (**left graph**) and from Coil B to Coil A (**right graph**). Each coil voltage was connected in series and rectified.

Voltages of Coil A and Coil B are subtracted (instead of summed) since they have opposite signs (refer to Figure 5). The subtracted 0.3 volts is the two-diode forward voltage drop in the bridge rectifier. Figure 7 shows the calculated voltage (grey line) and experimentally measured voltage (red line) of the output, within a freefall movement from Coil A to Coil B (left graph) and from Coil B to Coil A (right graph). There is a good agreement with the calculated and measured voltage (red and grey lines). Additionally, there is a good symmetry between the movements in both directions (left graph and right graph).

#### 4.1.2. Coils in Series after Rectification

The voltage values obtained from the freefall test, Figure 5, were used to calculate the output voltage of the system, where each coil voltage is rectified and then coils are connected in series. The output voltage was calculated with the test from Coil A to Coil B and vice versa. Van is the rectification (absolute voltage value) for coil (2), and V*bn* is the rectified output voltage in coil B (3). V*n*, the output voltage, is the sum (series) of both voltages (4). A forward voltage drop of 0.3 V was considered in the two diodes of the rectifier.

(2) $$V_{an}=\{\begin{array}{c} \;\;\;\;\;\;\;\;\;\;\;\;\;\;\;\;\;\;\;\;\;\;\;\;0,\;\;\;\left|V_{CoilA}\right|<0.30\\ \left|V_{CoilA}\right|-0.30,\;\;\;\left|V_{CoilA}\right|\geq 0.30 \end{array}$$

(3) $$V_{bn}=\{\begin{array}{c} \;\;\;\;\;\;\;\;\;\;\;\;\;\;\;\;\;\;\;\;\;\;\;\;0,\;\;\;\left|V_{CoilB}\right|<0.30\\ \left|V_{CoilB}\right|-0.30,\;\;\;\left|V_{CoilB}\right|\geq 0.30 \end{array}$$

(4) $$V_{n}=V_{an}+V_{bn}$$

**Figure 8:**
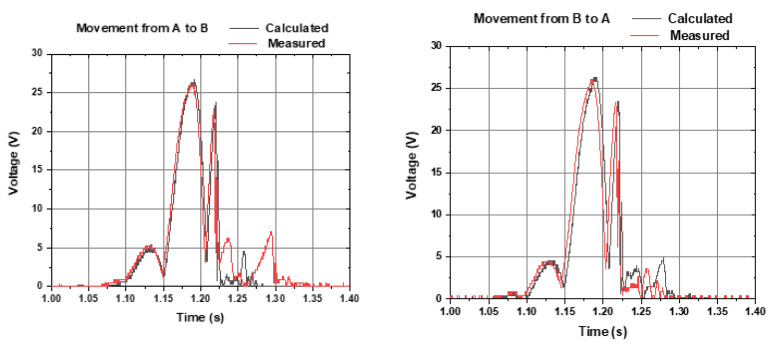
Calculated voltage (grey line) and experimentally measured voltage (red line) of the output, within a freefall movement from Coil A to Coil B (**left graph**) and from Coil B to Coil A (**right graph**). Each coil voltage was rectified and connected in series.

**Figure 9:**
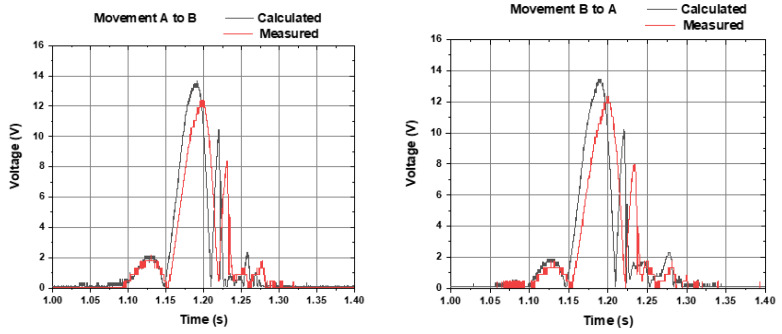
Calculated voltage (grey line) and experimentally measured voltage (red line) of the output, within a freefall movement from coil A to coil B (**left graph**) and from coil B to coil A (**right graph**). Coils were connected in parallel, followed by rectification.

Figure 8 shows the calculated voltage (grey line) and experimentally measured voltage (red line) of the output, within a freefall movement from Coil A to Coil B (left graph) and from Coil B to Coil A (right graph). Small differences are noticed from the results of Figure 8 (series before rectification) and Figure 9 (series after rectification), to be discussed further.

#### 4.1.3. Coils in Parallel Followed by Rectification

The two coils were connected in parallel, and the obtained voltage rectified by the full-wave rectifier. Equation (5) calculates the expected output voltages. Considering the results from Figure 5, the polarity of Coil A was inverted to obtain a higher voltage output when connected in parallel with Coil B.

(5) $$V_{n}=\{\;\;\begin{array}{c} \;\;\;\;\;\;\;\;\;\;\;\;\;\;\;\;\;\;\;\;\;\;\;\;\;\;\;\;\;\;\;\;\;\;\;\;\;\;\;\;\;\;\;\;\;\;\;\;0,\;\;\;\left|\left(-V_{CoilA}+V_{CoilB}\right)/2\right|<0.30\\ \left|\left(-V_{CoilA}+V_{CoilB}\right)/2\right|-0.30,\;\;\;\left|\left(-V_{CoilA}+V_{CoilB}\right)/2\right|\geq 0.30 \end{array}$$

V*n* is the output voltage value, V*CoilA* and V*CoilB* are output voltages from Coil A and Coil B, as presented in Figure 5, and 0.3 volts is the diode forward voltage drop.

Figure 9 shows the theoretical results calculated from the experimental result on each coil from Figure 5, and the experimental results from the average of six tests performed in freefall for each of the coils.

#### 4.1.4. Coils in Parallel after Rectification

Each coil voltage is rectified, and the total output voltage is obtained, connecting both rectified voltages in parallel. The voltage values obtained from the freefall test, Figure 5, were used to calculate the output voltage of the system. V*an* is the absolute value for Coil A (Equation (2)) and V*bn* (Equation (3)) is the absolute value for Coil B, with a drop voltage of FWBR of 0.3 V. The parallel connection after rectification is obtained theoretically by considering the maximum absolute value of Coil A or Coil B, in Equation (6).

(6) $$V_{n}=Max\left(V_{an},V_{bn}\right)$$

**Figure 10:**
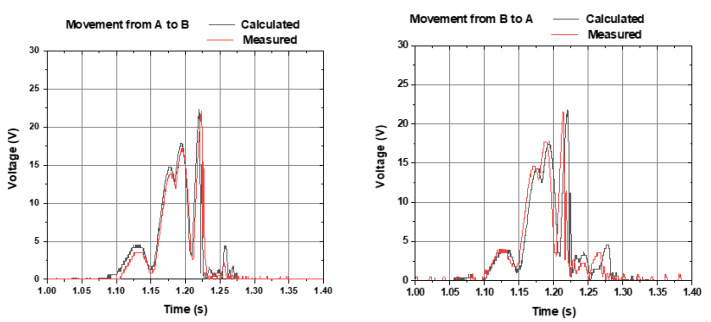
Calculated theoretical voltage (grey line) and experimentally measured voltage (red line) of the output, within a freefall movement from Coil A to Coil B (**left graph**) and from Coil B to Coil A (**right graph**), with coils parallelized after rectification.

Figure 10 shows the calculated theoretical voltage (grey line) and experimentally measured voltage (red line) of the output, within a freefall movement from Coil A to Coil B (left graph) and from Coil B to Coil A (right graph).

### 4.2. Comparison of Results

By comparing the theoretical and experimental graphs from previous figures, the best connection configuration can be selected. Table 2 presents the maximum voltage, measured or calculated, from the presented four configurations, series or parallel, with rectification before or after a combination of coils and with movements in both directions.

However, the maximum voltage, as presented in Table 2, is not sufficient to evaluate the best configuration. The battery charges only when the voltage is above 4.0 V. An effective LEG should provide voltages above 4.0 V for longer periods. The total amount of time that voltages are above 4.0 V can be calculated with Equation (7).

(7) $$\mathrm{Tot}=\overset{n}{\underset{n=4.1}{\sum }}\left(\left(CoilAn\right)A\to B+\left(CoilBn\right)B\to A\right)$$

The Tot is the total time when the generator can charge the battery of the two sets of values of the movement, Coil A to Coil B (CoilAn) and Coil B to Coil A (CoilBn). The total time calculation is presented in Table 3.

### 4.3. Output Power

The series connection, after rectification (Section 4.1.2) is used in the following characterization tests, as previous results demonstrated that this configuration maximizes battery charging. In the following tests, a load is applied in the generator, a resistive load, a battery or capacitor. A load allows the generator to produce output electric current and power, and consequently, mechanical energy is removed from the magnet movement. This energy removal slows the magnet movement due to a drag force caused by induction in the coils, and the slower movement reduces the output voltage generated until an equilibrium is found. In the test from the previous section, the only voltage was considered, without current flow, so its effect on the output voltage was not considered. In the following characterization tests, the power output and energy are calculated for different loads and frequencies.

#### 4.3.1. Load Dependence of Output Power

**Figure 11:**
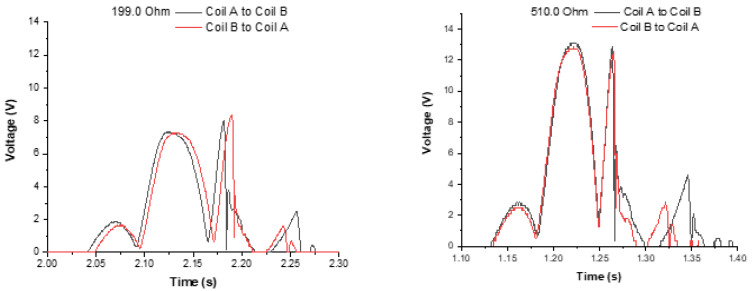
Measured output voltage in a freefall test, using several load resistors. Each graph shows voltage measured with movement from Coil A to Coil B and Coil B to Coil A. The (**left graph**) uses a load resistor of 199 Ω and the (**right graph**) a load resistor of 510 Ω.

With a set of load resistors, of several values, a set of six tests per resistor was performed, and the average values were considered to obtain the average power. Two examples of the measured voltages are presented in Figure 11, in a freefall test, using load resistors of 199 Ω and 510 Ω.

**Figure 12:**
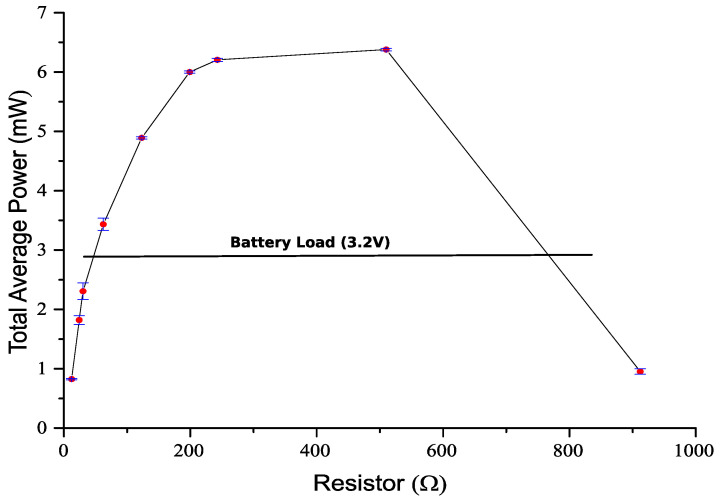
Load dependence of average power in freefall test. The line represents a constant voltage load of 3.2 V, equivalent to a lithium rechargeable battery. Mean values (dots) and mean ± standard deviation.

With the results of previous graphs, the average power is calculated by the Expression (8),

(8) $$P_{T}=\frac{{\left(Vrms\right)}^{2}}{R_{L}};\;V_{rms}=\sqrt{\frac{1}{N}\Sigma_{i\;=\;0}^{n}V_{i}^{2}}$$

where P*_T_* is the average output power, V*_rms_* is the RMS (root mean square) voltage, R_L_ is the load resistance, N is the number of samples and V*i* is the instantaneous voltage at each discrete instant i. The RMS voltage was calculated in all tests always considering the total time of 5.0 s (0.2 Hz), sampled at 313 µs period, resulting in *N* = 15,974 samples. The power was calculated separately, for the freefall tests of the magnet, from Coils A to B and from B to A, and the two values were summed to compute the power in a complete cycle of 5 s. Table 4 shows the average power for different loads, and Figure 12 shows graphically the output power as a function of the load resistor.

The maximum average power is obtained at resistances in the range 199 Ω to 510 Ω, despite the internal resistance of 125 Ω in each coil, and a total resistance of 250 Ω in a series configuration. An almost constant power can be obtained with loads from 199 Ω to 510 Ω. The maximum power is not obtained with a load resistor equivalent to the internal generator resistor for several reasons. First, the load current flowing in the coils causes a magnetic drag force in the magnet, which alters the magnet movement. In a parallel connection after the rectification circuit, the current that is extracted from each coil is not equally distributed, since it depends on the output voltage of each coil. Additionally, in a series connection after rectification, the output current can have a different direction in each coil, due to full-bridge rectification.

The generator is intended to charge a lithium battery presents an almost constant voltage (3 V to 4.1 V). To simulate this load, a 1F capacitor, pre-charged with 3.2 V, was connected as the load and measured the initial and final voltage of the capacitor to calculate the energy stored and average power. The energy stored can be calculated with Equation (9)

(9) $$P\cdot \Delta t=\Delta t=E=\frac{1}{2}C\left(V_{f}^{2}-V_{i}^{2}\right)$$

where P is the average power in Δt time interval, E is the energy, C is the capacitance value, V*_f_* is the final voltage, and V*_i_*, is the initial voltage of the capacitor. If the time Δt = 5 s is considered, in a 0.2 Hz movement (as in Table 4), the average power can be calculated.

In a freefall test, the voltage raised 4.13 mV from 3.216 V, and a power of 2.8 mW is calculated (voltage was measured in a series of 10 freefalls, from Coils A to B and from Coils B to A). This value is represented with a dashed line in Figure 12. As expected, the power obtained is below the maximum power with optimal load. To operate at the maximum power with optimum load, a maximum power point tracking (MPPT) circuit is required. For the expecting working power (6 mW in this test), such a circuit would present an efficiency that is usually below 70% (due to losses in the converter and energy to power the circuit), thus representing that only 4 mW could be used to charge the battery. The MPPT circuit still needs to be powered from this energy. The direct connection of the generator to the battery represents a huge simplicity in the circuit, and almost the same power can be delivered to the battery.

#### 4.3.2. Frequency Dependence of Output Power

**Figure 13:**
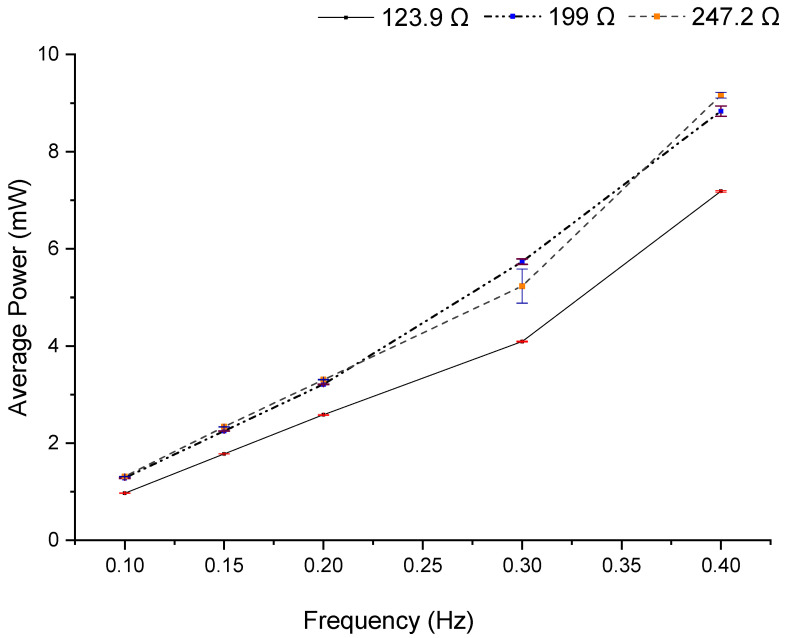
Average output power was measured in three different load resistance values, with movement frequency from 0.1 to 0.4 Hz. Mean values (dots) and mean ± standard deviation.

The following tests were performed at movement frequencies between 0.1 and 0.4 Hz since the system was designed to operate at these low frequencies. Tests were performed for all previous load resistances, but only the resistances/load with the highest average power are represented. The average power was calculated in all tests, always considering the total time of 50.0 s, with a sampling interval of 3.125 ms, resulting in 16,000 samples, using Equation (8). Figure 13 shows the average output power measured in three different load resistance values, with movement frequencies from 0.1 to 0.4 Hz.

The generator is intended to supply energy to submersed sensors. In the proposed configuration, up to eight sensors are considered. All eight sensors were characterized, and a mean power of 0.945 mW was measured in each sensor, using a voltage of 3.2 V (sensor datasheet shows data each sensor can be powered with an input voltage between 3 and 5 V).

**Figure 14:**
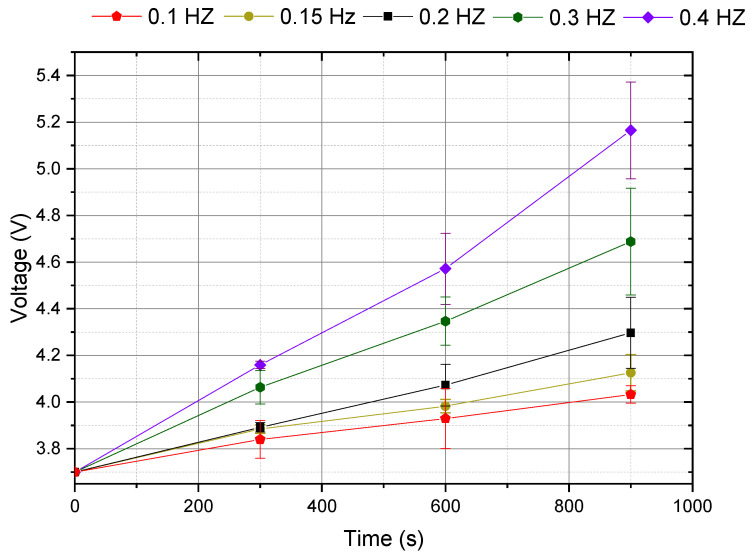
Capacitor voltage during a 900 s charge, with several movement frequencies.

Previous load tests were performed in a resistive load. However, in a real application, a lithium rechargeable battery (LiPO) or a supercapacitor is expected to be used. To ensure that the proposed system can supply energy at the desired frequencies, in addition to the load test with the resistors presented above (Table 3), tests are carried out to charge a 1 F capacitor to simulate a LiPO battery. A LiPO battery typically charges from 3 V (fully discharged) to 4.1 V (fully charged), with an almost linear voltage increase. For these, the initial capacitor voltage value is 3.7 volts, and tests were performed to increase the capacitor voltage above 4.1 V. Tests are made for frequencies 0.1 Hz to 0.4 Hz, and the final voltage was measured in the total time of 900 s. Figure 14 shows the capacitor voltage measured at the initial time of 300, 600 and 900 s.

**Figure 15:**
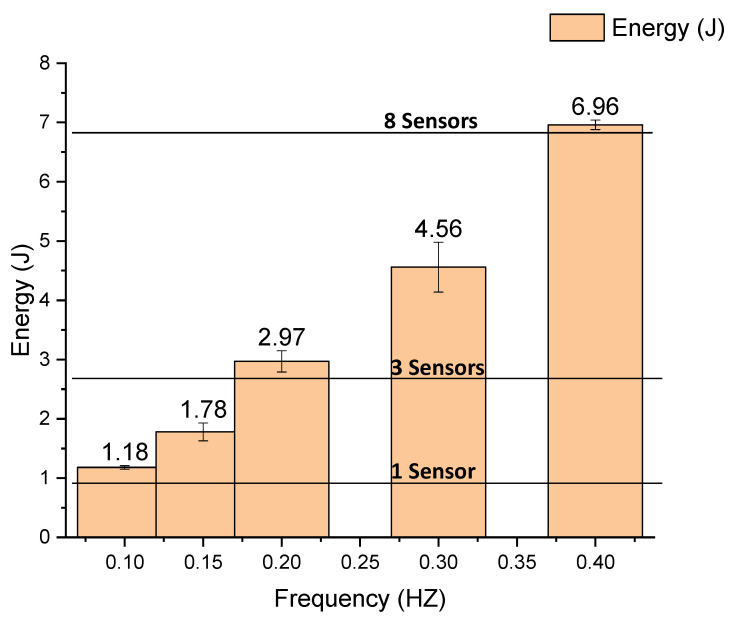
Energy stored in the capacitor for 900 s.

Considering the time-voltage graph of Figure 15, the stored energy in the capacitor can be calculated by Equation (9). The graph of Figure 15 shows the energy storage in the capacitor for 900 s.

For example, considering that the average power consumed by a sensor is 0.945 mW, then in 900 s, 851.43 mJ is consumed by each sensor. In this case, to supply power to at least three sensors in 900 s, the energy of 2.55 J is necessary. In Figure 15, it is possible to confirm that with the minimum frequency, it is possible to supply energy to the three sensors for which a 0.2 Hz movement is necessary; after subtracting 2.55 J for the three sensors, there is still 0.42 J remaining to charge an energy storage device. With a 0.4 Hz movement, eight sensors can be powered.

**Figure 16:**
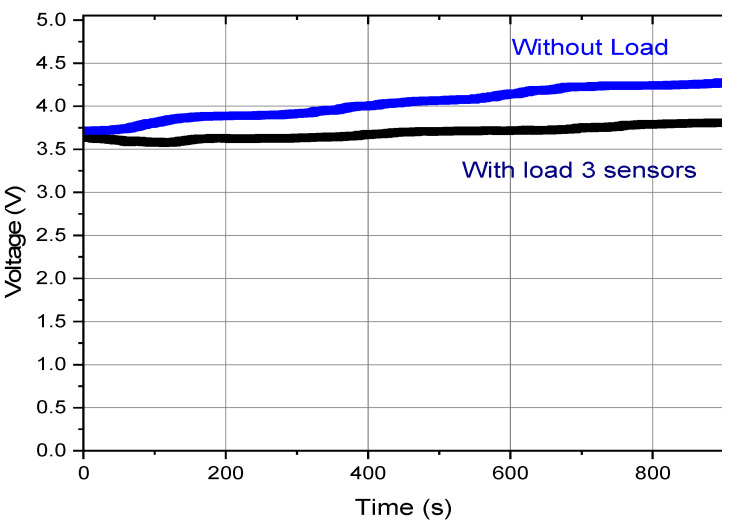
Capacitor voltage charging with 0.2 Hz movements, with 3 sensors load and without load.

To confirm these calculations, tests were realized with these sensors connected to the generator device. The LEG was operated at 0.2 HZ, and the same 1 F capacitor was connected in parallel with the three sensors load. Figure 16 shows the voltage evolution when charging the capacitor at 0.2 Hz, without a load and with the three sensors load. The capacitor was used to store the energy, but also as a simple mean to measure stored energy, as represented in Equation (9).

The charging procedure represented in Figure 15 was repeated six times, and the average results obtained are shown in Table 5. The capacitor initial voltage (V_i_) is always 3.7 V, and the final voltage (V_f_) was always measured at the total time of 900 s. Storage energy (E) was calculated with Equation (9), and the mean power delivered to the capacitor was calculated.

In Table 5, the first line (without load) energy represents the energy accumulated in 900 s, at a frequency of 0.2 Hz, without sensors connected (as previous Figure 14). The second line (with three sensors load) shows the energy stored in the capacitor for 900 s while powering three sensors. The experimental values show that in 900 s, it is possible to supply energy to three sensors and charge a battery with a surplus energy of 0.52 J, corresponding to a mean power of 0.6 mW. The experimental results (Table 6) validated the experimental results (Figure 14). The theoretical results point to 0.42 J remaining to charge the battery, versus 0.52 J measured experimentally, while powering three sensors and using the remaining energy to charge the capacitor.

Each sensor uses a mean power of 0.945 mW, representing 0.85 J during 900 s. The consumption profile of the sensor is described in Table 1. Considering the energy generated (presented in Figure 14) and experimental validation from Table 6, it is possible to predict how many sensors can be powered for several movement frequencies, as represented in Table 6.

In Table 6 are represented, from the left column to the right, the movement frequency, the total energy generated by the LEG (in a 900 s test) and respective power, the number of sensors that can be powered, the energy used to power the sensors, the excess energy that can be stored, and respective excess power.

According to the results obtained in Table 6, it is feasible to supply and charge a battery with this LEG configuration, depending on the frequency. It is important to note that at 0.4 Hz, the system can supply energy to eight sensors and still charge a battery with excess energy. It should be noted that the experimental results were obtained with sensors that consume an average of 0.28 J (in 300 s), as this sensor requires 300 µA current. Currently, these sensors are already optimized to use a current of 50 µA in sleep mode, which corresponds to the average energy consumption of 0.048 J in 300 s under the conditions presented in Table 1. With this consumed energy, it is possible to supply 48 sensors instead of 8; otherwise, excess energy is stored in the battery.

## 5. Conclusions

Several energy harvesting devices with different technology can be used on the ocean. For the intended application, it was decided to study and implement an electromagnetic energy harvesting device mainly due to the low cost of the electrical mechanisms. The proposed device does not require special electrical components, such as a tribolelectric generator or piezoelectric generators. The TEGs produce high voltage and small current, while the electromagnetic generator produces low voltage and is easy to convert to voltage to be uses in sensors. The TEGs need frequencies that are superior to 1 HZ to produce enough voltage, and for this application, the LEG has to supply energy with frequencies below 0.4 Hz. Another important point is the low load resistance of the TEGs that are in the order of MΩ. There are applications where it is preferable to apply TEGs with different electrical components, and in other applications, a combination of technologies of electromagnetic, piezoelectric, electrostatic, dielectric and triboelectric are necessary to apply. There will not be a universal solution.

A linear electromagnetic generator (LEG) was fabricated, using a magnet that moves along two coils, according to the current and water oscillations. This generator has no external moving parts, and it is intended to operate fully submerged. The LEG was fully characterized under several conditions, including powering oceanographic turbidity sensors.

By performing individual freefall tests on each coil, it is possible to predict the connections of coils to maximize the charge of a battery. Coil voltages were rectified in a full bridge rectifier and connected in series, for maximizing the charging time. The generator output power was evaluated with different resistive load values and a capacitive load to evaluate the effect of the load on the power output. An almost constant power (above 6 mW) was obtained with load resistors from 199 to 510 Ω. Since the generator is used directly connected to a battery, without a maximum power point tracking circuit, this flat power ensures the optimization of the power transferred to the battery. The flat behavior was attributed to drag forces caused by load currents. The use of an almost constant voltage load (capacitive load) has allowed us to verify that the circuit still operates at a useful power point (2.8 mW) when connected to a battery, and a maximum power point tracking circuit was not necessary.

Experimental results, both with resistive loads and a supercapacitor (simulating a lithium battery), were theoretically validated, with oscillating movements with frequencies from 0.1 to 0.4 Hz, as expected in typical ocean waves in the Atlantic Ocean. Generated power increases with frequency, from 1.31 mW at 0.1 Hz to 7.73 mW at 0.4 Hz.

The generator was used to power several turbidity sensors, and it was possible to supply power for up to eight sensors and still charge the battery with excess power, with a 0.4 Hz movement. The 0.1 Hz movement was also enough to power one sensor.

## Figures and Tables

**Table 1:** Energy consumed by each sensor.

Event	Time (s)	Power (mW)	Energy (mJ)
Sleep mode	299.99	0.96	287.99
Reading	0.01	320	3.2
Total	300.00	0.97	291.1

**Table 2:** The output maximum voltage is generated with different coil combinations.

Type Combination	Theoretical Values (V)		Experimental Values (V)	
	Coil A to Coil B	Coil B to Coil A	Coil A to Coil B	Coil B to Coil A
Series followed by rectification	27.10	26.62	26.19	25.31
Series after rectification	26.80	26.32	26.06	26.06
Parallel followed by rectification	18.13	17.80	22.08	21.64
Parallel after rectification	22.39	21.78	21.20	22.80

**Table 3:** Total time with generator voltage is above 4 V, with different coil combinations.

Connection Configuration	Total Time (ms)
Series followed by rectification	145.6
Series after rectification	202.4
Parallel followed by rectification	123.3
Parallel after rectification	188.3

**Table 4:** Average power vs. load in freefall test.

Resistor (Ω)	P_AB_ Coil A to Coil B (mW)	P_BA_ Coil B to Coil A (mW)	P_T_ One Cycle (A + B) (mW)
12.1	0.51	0.31	0.82
24.1	0.86	0.96	1.82
30.2	1.11	1.21	2.32
62.2	1.78	1.66	3.44
123.9	2.44	2.45	4.89
199.0	2.95	3.04	6.00
242.7	3.15	3.06	6.20
510.0	3.23	3.15	6.38
912.0	0.48	0.47	0.95

**Table 5:** Energy stored during 900 s at 0.2 Hz movements, and respective mean power.

	Vi (V)	Vf (V)	Energy (J)	Power (mW)
Without load	3.71	4.44	2.97	3.3
With 3 sensors load	3.71	3.85	0.52	0.6

**Table 6:** Number of sensors powered for each movement frequency. The energy was calculated for 900 s.

Frequency f (Hz)	Total Generated Energy E(J)	Total Power P_*T*_ (mW)	Maximum Number of Sensors N	Energy Used to the Sensors E (J)	Excess Energy Stored ∆E (J)	Excess Power ∆P (mW)
0.1	1.18	1.31	1	0.85	0.33	0.37
0.15	1.78	1.98	2	1.70	0.08	0.09
0.2	2.97	3.30	3	2.55	0.42	0.47
0.3	4.56	5.07	5	4.25	0.31	0.34
0.4	6.96	7.73	8	6.80	0.16	0.18

## Data Availability

Not applicable.
